# Evidence-based and guideline-concurrent responses to narratives deferring HCV treatment among people who inject drugs

**DOI:** 10.1186/s12954-019-0286-6

**Published:** 2019-02-11

**Authors:** Ellen Childs, Sabrina A. Assoumou, Katie B. Biello, Dea L. Biancarelli, Mari-Lynn Drainoni, Alberto Edeza, Peter Salhaney, Matthew J. Mimiaga, Angela R. Bazzi

**Affiliations:** 1 Department of Health Law, Policy and Management, Boston University School of Public Health, Boston, MA USA; 2 Section of Infectious Diseases, Department of Medicine, Boston University School of Medicine, Boston, MA USA; 3 Center for Health Equity Research, Brown University School of Public Health, Providence, RI USA; 4 Departments of Behavioral and Social Health Sciences and Epidemiology, Brown University School of Public Health, Providence, RI USA; 5 The Fenway Institute, Fenway Health, Boston, MA USA; 6 Evans Center for Implementation and Improvement Sciences, Boston University School of Medicine, Boston, MA USA; 7 Center for Healthcare Organization and Implementation Research, Edith Nourse Rogers Memorial Veterans Hospital, Bedford, MA USA; 8 Department of Psychiatry and Human Behavior, Brown University Alpert Medical School, Providence, RI USA; 9 Department of Community Health Sciences, Boston University School of Public Health, 801 Massachusetts Ave, 442e, Boston, MA 02118 USA

**Keywords:** HCV infections, Substance abuse, Intravenous, Risk factors

## Abstract

**Background:**

Hepatitis C virus (HCV) infection is increasingly prevalent among people who inject drugs (PWID) in the context of the current US opioid crisis. Although curative therapy is available and recommended as a public health strategy, few PWID have been treated. We explore PWID narratives that explain why they have not sought HCV treatment or decided against starting it. We then compare these narratives to evidence-based and guideline-concordant information to better enable health, social service, harm reduction providers, PWID, and other stakeholders to dispel misconceptions and improve HCV treatment uptake in this vulnerable population.

**Methods:**

We recruited HIV-uninfected PWID (*n* = 33) through community-based organizations (CBOs) to participate in semi-structured, in-depth qualitative interviews on topics related to overall health, access to care, and knowledge and interest in specific HIV prevention methods.

**Results:**

In interviews, HCV transmission and delaying or forgoing HCV treatment emerged as important themes. We identified three predominant narratives relating to delaying or deferring HCV treatment among PWID: (1) lacking concern about HCV being serious or urgent enough to require treatment, (2) recognizing the importance of treatment but nevertheless deciding to delay treatment, and (3) perceiving that clinicians and insurance companies recommend that patients who currently use or inject drugs should delay treatment.

**Conclusions:**

Our findings highlight persistent beliefs among PWID that hinder HCV treatment utilization. Given the strong evidence that treatment improves individual health regardless of substance use status while also decreasing HCV transmission in the population, efforts are urgently needed to counter the predominant narratives identified in our study. We provide evidence-based, guideline-adherent information that counters the identified narratives in order to help individuals working with PWID to motivate and facilitate treatment access and uptake. An important strategy to improve HCV treatment initiation among PWID could involve disseminating guideline-concordant counternarratives to PWID and the providers who work with and are trusted by this population.

## Introduction

Hepatitis C virus (HCV) infection continues to be endemic among people who inject drugs (PWID) in the United States (US), where the infection prevalence among PWID is estimated at 55.2% [1]. HCV is most efficiently spread through intravenous transmission, and in the context of the ongoing opioid epidemic, the number of HCV infections is continuing to rise [2]. The effectiveness of HCV treatment has improved dramatically with the introduction of direct-acting antiviral therapies, which are more effective and have fewer side effects than older treatments [3]. In addition to improving individual health and preventing the onset of costly end-stage disease, public health and medical guidance panels advocate for treating PWID to prevent onward HCV transmission and ultimately eliminate HCV in the population [4–7].

Intransigent concerns related to poor treatment adherence, drug relapse, and reinfection among substance-using patients, which are not supported by available data, persist [8]. Some of these concerns may stem from recent changes in treatment. Previous treatments involving interferon often involved adverse side effects (e.g., irritability, insomnia, psychiatric symptoms, and lack of appetite), heavy pill burdens, and lengthy treatment timeframes that contributed to poor adherence and treatment dropout [9, 10]. Studies of newer HCV treatments indicate fewer adverse side effects and greater adherence among PWID [11], but addiction-related stigma and so-called “sobriety requirements” restricting HCV treatment access or delaying initiation for patients with current or recent substance use endure [8, 12, 13]. Despite evidence of the efficacy of direct-acting antiviral therapy in curing HCV among those with current substance use, HCV treatment rates among PWID lag behind those of other groups [14].

The scientific literature related to prevention, care, and treatment of HCV among PWID focuses on patients’ progress along the continuum of HCV care from initial screening to eventual cure and identifying new ways to reach PWID in different settings with innovative interventions. Previous literature has identified particular barriers that PWID experience along this entire continuum, including receiving unclear explanations of a positive HCV test, the implications of the diagnosis, or what steps to take after initial testing; inadequate referral systems to treatment; and unclear explanations of disease progression or treatment recommendations from medical providers after liver testing [15–20]. A recent qualitative study with young PWID in the US Northeast also found perceived deservingness and need of treatment, as well as negative interactions with medical providers, to be additional disincentives surrounding HCV treatment [17]. An additional complication is that for those on Medicaid, requirements and restrictions for HCV treatment vary by state, with many states failing to provide guideline-concurrent HCV care by restricting treatment to those with higher liver damage and enforcing sobriety or prescriber restrictions [21].

While suboptimal HCV treatment utilization among PWID is often explained in terms of reasons for delaying treatment, previous studies have not directly juxtaposed PWID narratives to specific evidence-based data or treatment recommendations. In the current study, we summarize common narratives used by our sample of PWID to explain delayed HCV treatment and then compare these narratives to current medical literature and treatment guidelines to identify discrepancies and areas for improved patient and provider education and communication. Rather than portraying PWID as “misinformed,” our intention is to juxtapose persistent narratives, which regardless of their factuality are grounded in lived experiences and influence the ways in which people relate to the world, with evidence-based guidelines. Our objective is to equip health, social service, and harm reduction providers and other stakeholders working with PWID with current information to guide conversations and interventions to promote PWID engagement in HCV care.

## Methods

### Study design and sample

Data analyzed for this paper originated from a qualitative study focused on access to pre-exposure prophylaxis (PrEP) for HIV prevention among PWID in Boston, MA, and Providence, RI [22, 23]. To recruit eligible PWID, our study team partnered with community-based organizations (CBOs) frequented by PWID in both cities (e.g., syringe service programs, drop-in HIV/HCV testing centers). Eligible PWID were 18 years of age or older, reported injecting drugs in the last month, and reported HIV-negative status. Following referral to the study by CBO staff, trained study personnel conducted eligibility screening with interested individuals and, if eligible, obtained verbal informed consent in private spaces within CBOs. The institutional review board of the Boston University Medical Campus approved all study protocols and allowed a waiver of documentation of written consent by the participants to protect confidentiality as it would be the only record linking the participant to the study.

### Data collection

Between October 2016 and October 2017, trained interviewers administered brief demographic and behavioral questionnaires and then conducted in-depth interviews with PWID in private spaces in CBOs. The brief questionnaire included one question related to HCV, which asked the participant “has a medical provider ever told you that you have Hepatitis C?” If yes, the participant was asked to specify when. Semi-structured interview guides explored substance use behaviors, healthcare utilization patterns, and the acceptability of HIV prevention methods including PrEP. The interview guide included 16 questions asking about overall health, access to care, and knowledge and interest in specific HIV prevention methods, specifically PrEP. The qualitative interview guide was developed with the primary research questions related to PrEP knowledge and uptake among PWID in mind and was extended to include important related concepts from the literature (e.g., risk perception, healthcare access) and investigator experience. While HCV was not systematically investigated with a structured question in the interview guide, the question, “Tell me about your experience accessing health or other services for people who use drugs,” was followed by probes designed to help interviewers explore experiences with specific types of health services including HCV care, emergency services, drug treatment, and harm reduction services. Thus, although the interview guide was not developed to explicitly explore HCV treatment experiences or beliefs in an in-depth manner, due to the prevalence of HCV in the study population, when asked about healthcare utilization, HCV was discussed by most participants. Interviews, which lasted approximately 45 min, were audio-recorded and transcribed by a professional transcription company and reviewed by the study team for accuracy.

### Data analysis

The study team implemented a collaborative codebook development and coding process involving several steps [24, 25]. First, six research team members (including three investigators and three trained qualitative research assistants) read selected transcript excerpts independently to inductively generate potential codes and definitions based on topics of interest (i.e., key domains and questions from interview guides). We discussed potential codes and developed a preliminary codebook that team members then applied to another set of excerpts independently. We compared code application, discussed and resolved discrepancies, and modified the codebook for application to another set of transcripts. Through additional rounds of this process, we refined the codes and definitions until reaching consensus. Three analysts then used NVivo (QSR International Pty Ltd., version 11, 2017) to apply final codes to transcripts. HCV was first identified through initial team coding discussions as an important topic and assigned a code in the codebook. Following the application of codes in NVivo, further analysis for this paper involved close reading of data coded for HCV to identify key narratives surrounding HCV infection and treatment.

In order to explore narratives surrounding HCV within the broader at-risk PWID population, we did not exclude any participants from the qualitative analysis based on self-reported HCV status. Perceptions of risk of HCV were discussed by both participants who reported being HCV-infected and HCV-uninfected; however, the final analysis included perceptions and narrative related to HCV treatment, which only HCV-infected individuals discussed. During interviews, participants described being tested for HCV, being aware of HCV treatment, discussing HCV treatment with their medical providers, or completing preliminary liver testing. However, none described ever initiating HCV medication.

## Results

HCV was a common health problem among the 33 PWID participants in our sample, with 26 [79%] reporting ever being diagnosed with HCV (see Table 1 for additional sample characteristics, both of the entire sample and with just those who reported an HCV diagnosis). Experiences of treatment emerged through the qualitative interviews. Based on participants’ perceptions of HCV treatment, we identified three predominant treatment-related narratives: (1) lacking concern about HCV being a serious or urgent health threat and thus being uninterested in treatment; (2) recognizing the importance of treatment, but nevertheless deciding against initiating it; and (3) perceiving or experiencing that clinicians or insurance companies would recommend against treatment and thus not taking steps to initiate it. These participant narratives are expanded upon in the sections below.

**Table 1:** Characteristics of people who inject drugs (*n* = 33) and report having been diagnosed with HCV (*n* = 26)*

	Overall *n* (%)	Sample with HCV *n* (%)
Socio-demographics		
City		
Boston	16 (48%)	15 (58%)
Providence	17 (52%)	11 (42%)
Age in years; median (interquartile range)	36 (32–48)	36 (30–44)
Race (categories are not mutually exclusive)		
American Indian or Alaska Native	3 (9%)	2 (8%)
Black or African American	7 (21%)	4 (15%)
White	22 (67%)	21 (81%)
Other	5 (15%)	3 (12%)
Ethnicity: Hispanic/Latino	8 (24%)	6 (23%)
Gender		
Male	18 (55%)	14 (54%)
Female	13 (39%)	12 (46%)
Transwoman	1 (3%)	0 (0%)
Genderqueer	1 (3%)	0 (0%)
Sexual orientation		
Heterosexual or “Straight”	21 (64%)	16 (62%)
Bisexual	8 (24%)	7 (27%)
Homosexual or Gay	4 (12%)	3 (12%)
Sexual health and substance use behaviors		
Diagnosed with HCV, ever	26 (79%)	26 (100%)
Any distributive or receptive syringe sharing, past month	21 (64%)	18 (69%)
Number of people with whom participant shared injection paraphernalia (cookers, cottons, rinse water), past month		
0	12 (36%)	8 (31%)
1–2	10 (30%)	9 (35%)
≥ 3	11 (33%)	9 (35%)

*May exceed 100% when categories were not mutually exclusive

### “We don’t give a shit about Hep C:” lacking concern about HCV and being uninterested in treatment

A common narrative of participants reflected their lack of concern about HCV, with participants perceiving that HCV was not serious or did not pose a major threat to their health. Participants described having especially low concern about risk behaviors and contracting HCV when they were experiencing withdrawal symptoms, which was a frequent, if not daily, experience. As one 43-year-old man from Boston who reported having HCV explained:

We all know well and good that there’s more than one strain of Hep C, but if me and you were out there and say my needle broke or I did not have one or you only had one you are already using, I’d say, “Hey, I gotta use your needle.” And you’d be like, “Well, uh, you got Hep C?” And it’s just—[LAUGHS]—it’s almost as if you did not say anything at all because you are just like, “Yeah, whatever. Give me it.” You know? does not cross your mind at all.

In contrast, participants feared the perceived higher severity of HIV infection and reported discussing HIV before sharing injection equipment. A 33-year-old woman from Providence compared the threat of HCV to that of HIV, saying,

Like, “you don’t have AIDS? All right, yeah, give me your needle.” [LAUGH] That’s like the first thing that people say to me ‘cause they always want my needle and I’m like, look, well, I got-I got Hep C and they’re like, oh, we don’t give a shit about Hep C, yeah. It’s all right, you got Hep C, kinda brushin’-like-like sweepin’ it under the rug. But, HIV is like the elephant the room.

While participants perceived and reiterated the serious health consequences they associated with HIV, the broader and long-term health risks associated with HCV were not discussed in interviews. With the already high prevalence of HCV in this population and the lack of a perceived immediate health impact, HCV infection was not viewed as a serious medical condition requiring treatment.

### “You should probably wait to get the treatment:” recognizing the importance of HCV treatment but providing reasons against initiating it

Another narrative that was common in our sample involved knowing one’s chronic HCV infection status but providing several specific reasons for not seeking treatment. First, despite their known HCV diagnosis, some of these participants had very low knowledge of or interest in the new direct-acting antiviral therapies. For example, when asked if her physician had explained the HCV treatments that were now available, a 25-year-old woman from Boston responded, “No…They haven’t told me about them, and I mean I haven’t asked either, really…whatever, it’s a two-way street.”

Some participants who were generally aware of HCV treatment were concerned about side effects or had misconceptions about the new treatment. These individuals had heard from peers who had undergone months of older interferon treatment with side effects that were described as worse than the HCV infection symptoms. Concerns about side effects were particularly important in light of the frequency with which participants were already experiencing drug withdrawal symptoms and other illnesses. As this 35-year-old woman from Boston explained,

I hear it makes you really sick. I get sick on my own easily. I have a really weak immune system, so, um, anything that’s going to make me sick, I do not-I am not really looking forward to doing.

Another participant, a 33-year old woman from Providence, was concerned about older treatments she had heard about that required injections. Since she was entering recovery, she was trying to avoid drug-related triggers, which for her included needles:

I wanted to [start hepatitis C treatment], but they did not have the pill out last time. It was just injections and I did not want…the needle. So that’s why I blew it off. But now, I guess there’s a new pill form and it’s not as many weeks or something. So I have avoided the doctor for a while. I mean, like I have not had my period in over a year so I need to… get clean and take care of myself. That’s what I need to do.

Although this participant expressed awareness of new treatments being available, she provided additional reasons for not initiating HCV treatment, including not wanting to access medical care and prioritizing recovery and other health concerns first.

A 29-year-old male from Providence provided two different reasons for delaying treatment: waiting for “new” medications that he did not believe were available yet and not believing that his disease was bad enough to require immediate treatment:

I am probably gonna do that shit, the medication or whatever. There’s a new one. I want to wait for that one, I guess….Right now, I do not know what it [my HCV viral load] is right this second. It’s probably not…it’s not high. I know it’s not high. It’s low. It’s like undetectable, basically…it’s really nothing crazy.

Other participants explained that they did not want to start treatment until after they had stopped using drugs, believing that they would be quickly re-infected or infected with another genotype of HCV because they were still using drugs. As a 24-year old woman from Boston said:

So if you know that you are not done running, which I am definitely not done, if we are being honest, I am not done…So if you know you are gonna go back out there and keep using, you should probably wait to get the treatment. Because you do not know if maybe you are gonna end up sharing with someone with Hep C again.

While participants with the narratives described above generally believed that treatment was important, they had put off initiating treatment for a variety of reasons including low knowledge about new treatments, concerns or misconceptions about side effects, prioritizing recovery, avoiding medical care, and waiting for improved treatments that they believed were not available yet.

### Perceiving or experiencing clinician or insurance company recommendations against HCV treatment

Some participants reported seeking out HCV treatment, but none had completed treatment, for which they provided two interrelated reasons. First, participants reported that clinicians opted to postpone treatment because, in their medical opinion, participants were not yet appropriate candidates for therapy. Some participants described being told by clinicians that patients should be “clean” (i.e., not using drugs) for 6 months or more and in “more stable” living situations or environments before initiating HCV treatment. One 35-year-old woman from Boston hesitated while explaining the reason why medical providers had delayed starting her on HCV treatment, saying, “they were waiting for me to be clean for a little bit.” Other participants reported that clinicians delayed starting their treatment because they were infected with multiple HCV genotypes, making treatment more difficult or complicated. A 35-year old woman from Boston related her multiple genotypes of HCV and unstable living environment when describing her doctor’s decision to delay her treatment:

I have two different strands so it would be a lot more complicated. Even with the treatment that’s out right now, I would have to take a different pill, so [the doctor] wants to wait until I get a stable environment.

Related to clinician concerns about stability in housing and recovery and related assumptions about adherence challenges, some participants also reported that clinicians did not view them to be appropriate candidates for HCV treatment because their disease stage was not “bad enough” to warrant treatment. As a 48-year old man from Providence reported:

Well, I am not sick. The doctor told me that my liver’s not inflamed. There’s no need to get treatment. But he said if you continue drinking and doing drugs, then your liver is going to get inflamed and you are going to have to get treatment. So right now, I am good right now. If I continue drinking alcohol and doing the dirty stuff too, it’s going to get worse.

PWID narratives related to HCV could thus justify delayed treatment if the disease was “too complicated” or “too mild.” It is important to note that participants often described clinicians’ opinions as primary sources of information influencing their narratives.

Second, several participants emphasized the significant cost of HCV treatment, which they believed helped justify clinicians’ expectations that they be “clean” before starting treatment. As a 43-year old man from Boston said:

I was going through all the process and everything, I just gotta get six months or more clean under my belt to get the pills ‘cause I guess it costs $90,000 for the cycle. [Interviewer: Did someone tell you that?] Yes…[at] different places, but I was seeing a Hep C doc in [hospital].

Participants also believed that Medicaid would only pay for HCV treatment once, creating a sense that people were given “one chance” at treatment, which further justified their or their clinicians’ desires to wait until not using drugs to avoid “messing up” and becoming re-infected. As a recently diagnosed 24-year-old woman from Boston explained:

So if you go through MassHealth [to] get the treatment and you end up being clear [so] you do not have Hep C anymore, and then you go back out on another two-year run [using drugs], and end up in the same situation, where I am with somebody again and I am dope sick and I wanna use the needle, and I do not care, and I am being reckless, and I just want it now, then you give yourself Hep C all over again, and MassHealth is gonna be like, “We don’t wanna pay for that again.” So it might be smarter to wait till I am in a stable situation to get the treatment. That’s what they were saying at detox, and it’s probably a good point, if they are only gonna pay for it once…‘Cause I guess it’s really expensive. For like a bottle of a prescription, it was like 90 K or something crazy. That’s insane.

This narrative reflects the influence of information about medication access and cost—whether accurate or not—on treatment initiation in this stigmatized population.

These participant narratives reinforce a larger idea about clinicians and insurance companies not wanting to treat HCV in PWID, identifying inaccurate information related to delaying treatment when HCV is complicated or not severe enough, and pervasive concerns surrounding HCV treatment costs. Importantly, we have no way to corroborate the participants’ accounts with what the clinicians themselves said to the participants or to identify the type of clinician (e.g., primary care, emergency) who told them this information. But participants based these narratives on information they received from clinicians, other service providers, and peers, highlighting potential audiences for training and educational initiatives, as discussed in the next section.

## Discussion

Current Infectious Diseases Section of America and American Association for the Study of Liver Diseases (IDSA/AASLD) guidelines recommend HCV treatment for PWID including individuals who are actively using drugs [6]. Strong evidence demonstrates that concerns about poor HCV treatment adherence among PWID are unfounded [14]. However, despite high prevalence of HCV infection in our sample of PWID in the US Northeast, no participants had initiated HCV therapy (though one individual described being in the pre-treatment testing phase). Our findings echo previous literature that inaccurate, non-guideline-adherent narratives and explanations for deferring treatment persist in this population, which may stem in part from knowledge of previous treatments that were lengthy and difficult [10, 15–17]. By limiting treatment uptake, these inaccurate narratives prevent the improvement of individual health and enable ongoing transmission of HCV. Countering these narratives with accurate information is an important first step in improving HCV treatment uptake and outcomes among PWID.

### Countering the inaccurate narratives with guidelines and evidence-based practice

We identified common narratives involving explanations for why PWID avoid HCV treatment. Many sources of influence likely interact to shape these narratives, including conversations with friends or acquaintances who have undergone HCV treatment or know someone who has, patient interpretations of information provided by clinicians, descriptions of treatment from other health agencies, community-based organizations, or public service announcements, and rumors or word-of-mouth based in part on inaccurate media coverage of the disease. Importantly, these narratives often run counter to growing evidence and current guidelines on treating individuals with chronic HCV infection. Table 2 contrasts summaries of participant narratives from our study with relevant evidence and treatment guidelines to create a concise reference to help providers and other stakeholders working with PWID improve treatment uptake and general health in this population.

**Table 2:** Evidence-based responses to representative examples of common narratives by PWID in the US on reasons to delay HCV treatment

Narratives of PWID	Evidence	References
*I do not think my HCV is bad enough to warrant treatment now.* *I know I am infected with HCV, but I am not worried about it now.* *My doctor says my HCV is not bad enough to warrant treatment.*	• Curing all HCV-infected PWID of HCV benefits individual and overall public health. • Given the high rate of transmission of HCV through intravenous drug use, reducing HCV infection prevalence in the PWID population will reduce the overall epidemic • Some Medicaid programs use sobriety and prescriber restrictions to limit treatment access, which may deter physicians from discussing HCV treatment with patients with limited liver fibrosis; however, these practices run counter to current guidelines on treating HCV among PWID	[40, 13, 41]
*I am afraid of the side effects of HCV medication.* *I want to avoid drug-related triggers.*	• New direct-acting antiviral treatments are well-tolerated with limited side effects, even among individuals who are difficult to treat • New treatments do not require injections that could be triggering for individuals in recovery from drug use.	[14]
*I want to wait until I am done using drugs so I do not contract it again.* *My doctor wants me to be more stable before I start treatment.*	• PWID are adherent to HCV treatment and have low rates of reinfection • Combining HCV treatment with medication assisted treatment for opioid use disorder or harm reduction services (e.g., syringe exchange) can support PWID in completing HCV treatment • Curing all HIV-infected PWID of HCV benefits individual and overall public health.	[4, 42–46]
*I am unable to get treatment while I am inside detox, jail or prison.*	• HCV treatment availability in correctional settings varies, but research shows that it is feasible (though maintaining engagement in care post-release is a concern) and a growing number of facilities are providing therapy to incarcerated individuals {Beckman, 2016 #3713}	[47, 48]
*The treatment is very expensive, so the insurance company want me to be clean before I start it.* *Medicaid will only pay for HCV treatment once, so I need to be sure I am done using drugs before I start treatment.*	• Testing and treating PWID for HCV is cost-effective • Some states with known Medicaid reimbursement criteria limit treatment to those with advanced liver disease, and other states’ Medicaid reimbursement criteria require substance use screening and documentation for treatment; however, these limitations are not in line with current HCV treatment guidelines.	[49, 50, 8, 21, 13, 41, 7]

Specifically, Table 2 juxtaposes predominant representative narratives of PWID in the US with related evidence. In an attempt to make the table more concise and usable for people working with PWID, the narratives from the themes identified above are organized by the evidence to counter the narrative. For example, regardless of whether the participant reports that she wants to wait until she is more stable or that her doctor wants her to wait until she is stable, the current HCV guidelines and data are the same: curing all HCV-infected individuals will benefit overall individual and public health, and that concerns about medication adherence or risks of re-infection are unfounded (especially in combination with medication-assisted treatment and harm reduction). Individuals who work with PWID or health or social service providers could use this table to access current evidence and guidelines and formulate possible responses (i.e., counternarratives) or conversation points to help educate and encourage HCV treatment uptake among their clients and patients.

Based on our findings, we have several recommendations for the dissemination of these HCV treatment counternarratives. First, to ensure that provider responses to PWID narratives are accurate, it will be vital to ensure that health care professionals, community-based organization staff, drug treatment and detoxification center staff, and others who work with PWID living with HCV understand the evidence and how to communicate about it. In particular, these individuals will need communication strategies to counter predominant beliefs and misconceptions that are acceptable and accessible to the local communities in which they work. Limited and inaccurate knowledge surrounding medical care among PWID is not a new finding. Previous literature has indicated that misinformation related to the screening, diagnosis, and treatment of HCV may be related to low knowledge and motivation, perceived stigma of injection drug use, and past experiences of discrimination when seeking medical care [15, 17]. Studies of PWID identify low knowledge of HCV in general, and treatment in particular, as barriers for individuals to initiate treatment [15–17, 26]. PWID may also lack motivation to treat their HCV because drug use takes precedence or because they believe HCV infection is not serious [15, 26]. Provider communication strategies will require an understanding of not only the evidence but also the barriers that predominate in the communities in which they work.

Second, evidence-based interventions should be implemented to overcome barriers to HCV treatment identified in predominant, inaccurate narratives. Previous interventions have focused on training medical providers on current guidelines. Physician non-adherence to HCV screening guidelines is common, possibly indicating attitudinal barriers as well as gaps in knowledge [27, 28]. Interventions have used community-based clinics, substance use treatment clinics, and other more specialized hospital-based clinics to train medical providers on current guidelines [29–31]. One program in Ireland trained methadone providers to promote HCV screening and treatment for methadone patients, resulting in statistically significant difference in referrals to HCV care among general practitioners providing methadone treatment who had received the training than those who had not [32]. Future studies should examine the effectiveness of training for other types of providers who work with PWID, including staff at CBOs, syringe service programs, and drug treatment programs.

Interventions that target patient-derived barriers should also be adopted and adapted. Previous interventions have successfully targeted barriers to entering medical settings for HCV care by providing screening and services at locations that are convenient to PWID, including methadone clinics, drug treatment programs, detoxification centers [33–35], mobile medical clinics [30, 31, 36], and by using tele-medicine [37]. Other interventions have focused on engaging patients in clinical care to increase HCV treatment knowledge and motivation [38, 39]. Among Veterans Affairs HCV patients, individuals randomized into six 2-h self-management workshop sessions involving sharing information, developing problem-solving techniques, and developing, evaluating, and revising action plans had better knowledge, energy, and wellbeing than individuals randomized into the information-only control condition [38]. In a methadone clinic-based group intervention study, intervention arm participants receiving HCV screening, motivational-enhanced education and counseling, and case management were more likely to receive an HCV evaluation [39]. These interventions are promising to counter barriers to HCV treatment, but more research is needed to determine how to scale out the evidence-based interventions into new settings and systems. Despite the promise of these interventions, the prevalence and predominance of the inaccurate treatment narratives among PWID is concerning.

Our findings should be considered in light of several study limitations. First, although we attempted to purposively sample respondents to understand diverse perspectives, we recruited individuals in two urban, resource-rich areas of New England where CBOs and medical centers provide HCV, HIV, and comprehensive medical services including addiction medicine, infectious disease, and hepatology specialty services. Furthermore, the states in which this study was conducted, Massachusetts and Rhode Island, have been on the forefront of treating individuals with HCV and addressing substance use disorder, receiving an “A” grading from for HCV Medicaid policy [21]. Thus, our findings may not be generalizable to other geographic regions or settings without these resources or policy supports, where inaccurate narratives and other barriers to treatment could be even more prevalent and difficult to change. Second, HCV was not the primary focus of our original study on PrEP for HIV prevention among PWID. Nevertheless, HCV was a probe for a question about general healthcare utilization and emerged as an important topic for the majority of participants, and similar to our findings regarding PrEP, this study identified suboptimal knowledge and uptake of HCV treatment [22]. Additional research may be beneficial in systematically identifying local narratives to delaying HCV treatment and developing and testing intervention strategies to overcome barriers to care.

In summary, our findings highlight persistent, inaccurate narratives surrounding HCV treatment among PWID in the US that can be countered with updated information on evidence and guidelines. By exploring health and healthcare access of PWID, we uncovered three main reasons for delaying or forgoing HCV treatment despite the ongoing opioid epidemic and existence of highly effective HCV therapy. Many participants failed to view HCV as an important medical condition. Although curative HCV therapy was viewed as a helpful development, many PWID perceived that clinicians and insurance companies recommended delaying therapy until they stopped using drugs and lived in more stable environments. In response to these narratives, we provide evidence demonstrating the role of therapy in improving individual health (regardless of disease stage) and decreasing onward HCV transmission, as well as additional guideline-concordant information to encourage treatment initiation among PWID. It is our hope that this information can be used by health, social service, and harm reduction providers, people who inject drugs, and other stakeholders to dispel misconceptions and promote PWID engagement in HCV treatment and care.

## Abbreviations

CBOs: Community-based organizations
HCV: Hepatitis C
HIV: Human immunodeficiency virus
PrEP: Pre-exposure prophylaxis
PWID: People who inject drugs
US: United States
